# Rescaling of Distance Judgments With Geometric and Contextual Changes

**DOI:** 10.1002/hipo.70088

**Published:** 2026-03-10

**Authors:** Ernest Simons, Caswell Barry, Caroline Whyatt, Rebecca Knight

**Affiliations:** ^1^ Department of Psychology, Sport and Geography University of Hertfordshire Hatfield Hertfordshire UK; ^2^ Cell and Developmental Biology, Division of Biosciences University College London London UK

**Keywords:** distance perception, entorhinal cortex, spatial navigation, spatial processing, virtual reality

## Abstract

Grid cells have been identified in the entorhinal cortex of rodents and humans, as well as other mammals. In rodents, these “distance computing” neurons exhibit altered firing fields in response to environmental manipulations, including changes to geometry or specific contextual cues (e.g., color). The current study investigated whether these neurophysiological observations in rodents could predict human behavior in a distance judgment task under various environmental manipulations. Participants (*n* = 51) completed 22 trials involving distance traversal, memorisation, and distance replication across five experimental conditions: control (no manipulation), contextual manipulation (novel environment), and geometric manipulations (local expansion and contraction; global expansion and contraction). Results demonstrated that environmental expansions led to significant overestimations in distance judgments, consistent with rodent grid cell data. Global geometric manipulations yielded significant overestimations compared to the control condition. For the local manipulations, judgments were least accurate when made in the vicinity of the local manipulation. These behavioral patterns are consistent with localized deformations in spatial representations, as would be predicted from rodent grid cell studies. As hypothesized, changes to the environmental context (the novel environment condition) also resulted in significant distance overestimations. In conclusion, environmental manipulations influenced the accuracy of human distance judgments in a manner paralleling the firing field changes observed in rodent grid cells under similar environmental alterations. These findings demonstrate behavioral parallels between human distance estimation and rodent grid cell responses to environmental manipulations, suggesting possible commonalities in spatial processing across species.

## 1 Introduction

Animals are thought to construct a cognitive map of their environment within the hippocampal formation, enabling flexible navigation to remembered goals (O'Keefe and Dostrovsky 1971; Tolman 1948). This notion is supported by evidence that hippocampal lesions impair navigation to hidden goals in both rats and humans (Maguire et al. 2006). The neural basis of this cognitive map and its associated navigational computations likely comprises an array of spatially selective cells within the hippocampus and related regions. These include head‐direction cells, which signal facing direction (Butler et al. 2017; Taube et al. 1990); border cells, which relay boundary proximity (Alexander et al. 2020; Solstad et al. 2008); grid cells, which encode distance traveled through regularly periodic firing fields (Barry et al. 2007; Gu et al. 2018; Hafting et al. 2005); and place cells, which provide a sparse representation of self‐location (Jayakumar et al. 2019; Plitt and Giocomo 2021).

Grid cells, primarily associated with the medial entorhinal cortex (MEC) (Hafting et al. 2005), have attracted considerable theoretical attention due to their regular firing properties. These cells exhibit multiple firing fields arranged in a tessellated hexagonal pattern, which has been proposed to serve as a basis for integrating self‐motion cues and calculating heading vectors towards remembered goals (Banino et al. 2018; Burak and Fiete 2009; Burgess et al. 2007; Bush et al. 2015; McNaughton et al. 2006). While the grid pattern itself likely emerges from the integration of self‐motion signals, it is stabilized through associations with the sensory environment (Hardcastle et al. 2015). Thus, parametric manipulations to the geometry of familiar environments predictably deform grid cell firing patterns, suggesting an interplay between environmental cues and internal representations (Barry et al. 2007). Notably, the inherent structure of the environment also interacts with the regular structure of grid patterns, an effect which might express the available transitions afforded by environmental structure (Krupic et al. 2015; Stachenfeld et al. 2017; Stensola et al. 2015).

Environmental novelty also influences grid cell firing. In unfamiliar environments with altered contextual cues but identical geometry, grid cells exhibit an expanded scale and increased firing irregularity (Barry et al. 2012). This expansion attenuates as the environment becomes familiar, suggesting grid cells dynamically adapt to environmental novelty. Such plasticity may play a crucial role in distinguishing between similar environments and in forming new spatial memories or may be a means of maintaining efficient encoding of self‐location in the face of elevated spatial uncertainty (Towse et al. 2014).

Grid cells are not unique to rodents but are a common feature of the mammalian brain. In humans, evidence for grid‐like representations comes from both single‐cell recordings (Jacobs et al. 2013) and fMRI studies revealing six‐fold periodic modulation in entorhinal cortex activity during virtual navigation (Doeller et al. 2010). This “grid cell‐like representation” has been observed across various studies (Bellmund et al. 2016; Gönner et al. 2023; Horner et al. 2016; Moon et al. 2022) and, like rodent grid cells, can be disrupted by environmental barriers (Derdikman et al. 2009; He and Brown 2019). A behavioral study by Chen et al. (2015) demonstrated that manipulating environmental geometry in a virtual reality task led to distortions in distance judgments that appear to mirror the effects observed in rodent grid cells.

We have extended previous behavioral work (Chen et al. 2015), by manipulating both geometric and contextual cues during an object replacement task. Our study introduced several innovations: local geometric manipulations alongside global ones, assessment of environmental novelty effects by changing the context of the environment (color and texture of walls, floor and ceiling), and movement constraints to a single dimension. These modifications allowed for a more comprehensive evaluation of how environmental changes influence human spatial cognition. Our results demonstrated that geometric changes, as well as environmental novelty, led to significant overestimations in distance judgments. Notably, contextual manipulations produced larger effects than geometric manipulations. For geometric manipulations, global geometric manipulations produced larger effects than local ones. These findings suggest a possible link between grid cell‐like representations and human spatial behavior, bridging rodent neurophysiology and human cognitive mapping.

## 2 Materials and Methods

### 2.1 Participants

A total of 51 students (26 females and 25 males) from the University of Hertfordshire were obtained through an opportunistic and voluntary recruitment method. Ages within the sample ranged from 16 to 67 years with a mean age of 23.02 years (SD = 7.18). Participants all had normal or corrected‐to‐normal eyesight. All participants gave written consent and procedures were approved by the University of Hertfordshire ethics committee, with delegated authority (protocol number LMS PG/UH/00372).

### 2.2 Apparatus

Participants were seated in front of a desktop computer, approximately 40 cm from the screen, with the immersive virtual reality software Vizard (World Viz) loaded and displayed on screen. Within the environment, a first‐player character model was created to enhance the feelings of immersiveness. Participants could control the movement of this character by using the four arrow keys. The original environment was a 60 × 90 m room.

### 2.3 Design

**FIGURE 1:**
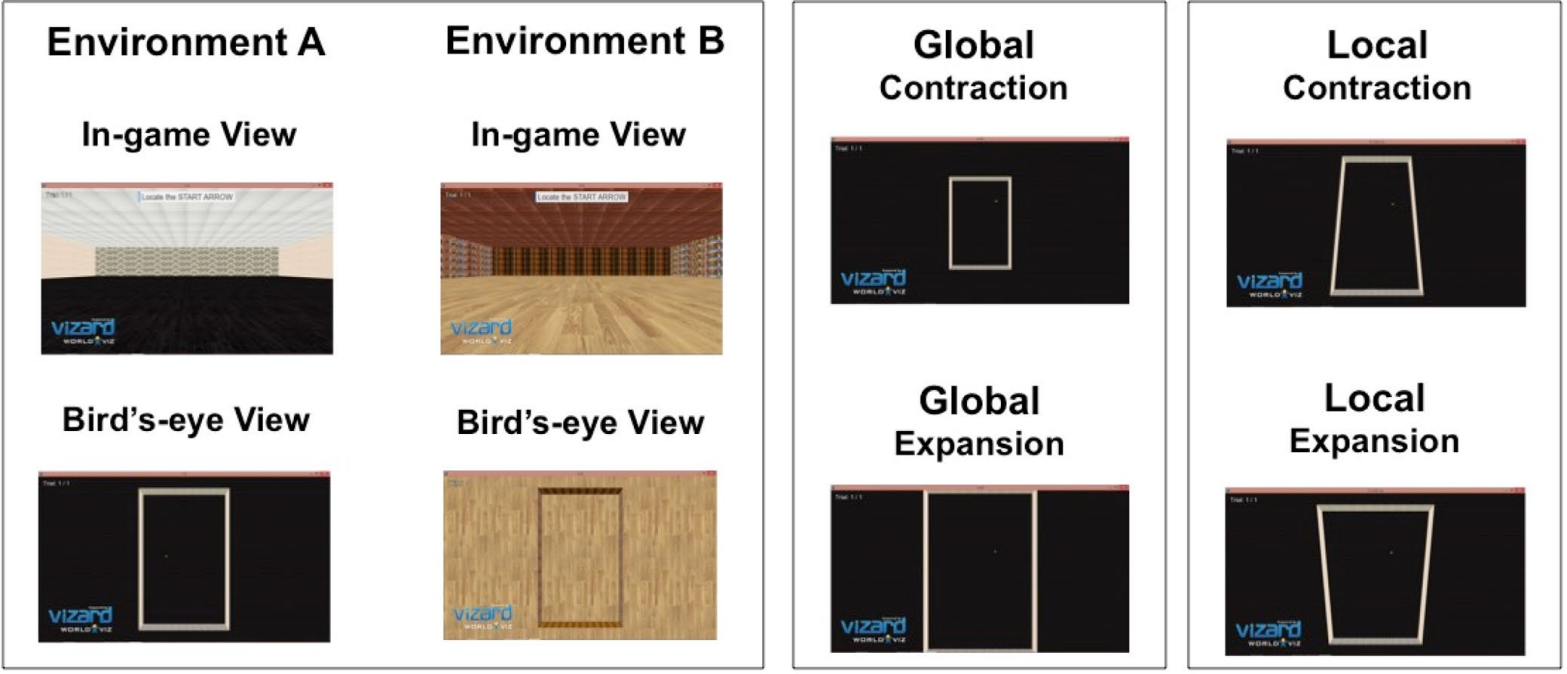
Schematic views of manipulations used in this study. *Left Panel*. An in‐game view and bird's eye view (with ceiling removed) of Environment A and B, used as either the original environment or novel environment, counterbalanced across participants. Thirty‐six participants saw Environment A as the original environment and Environment B as the novel environment. The remaining participants saw Environment B as the original environment and Environment A as the novel environment. *Middle Panel*. Global manipulations could occur in the horizontal and/or vertical dimensions of the environment; as a result, the newly created environment was enlarged parametrically in either the length, width or as seen in the figure, both. This resulted in six globally manipulated environments; Length expansion/contraction (60 × 120 m|60 × 60 m), Width expansion/contraction (80 m × 90 m| 40 m × 90 m) and Overall expansion/contraction (80 × 120 m| 40 × 60 m). *Right Panel*. Local manipulations consisted of alterations to the intersection where two boundaries met, that is, a corner. The manipulations resulted in the overall cuboid shape of the environment being truncated in two of the four corresponding corners. Four locally manipulated environments were produced: Local length expansion/contraction and local width expansion/contraction.

The study employed a within‐subjects design. In the retrieval phase, the original environment (presented in the encoding phase) underwent a number of manipulations (Figure 1). The first independent variable (IV) was the type of geometric manipulation employed (global vs. local). The second IV was the direction of manipulation (expansion or contraction). The third IV related to the geometric dimension that the manipulation was applied to (length, width, or both). Finally, the fourth IV manipulated the contextual rather than geometric features of the original environment (novel vs. original). The novel environment was identical in dimensions to the original environment but had different texturing applied to the walls, ceiling, and floor. The dependent variable was the accuracy of distance judgments measured in meters (m).

### 2.4 Procedure

**FIGURE 2:**
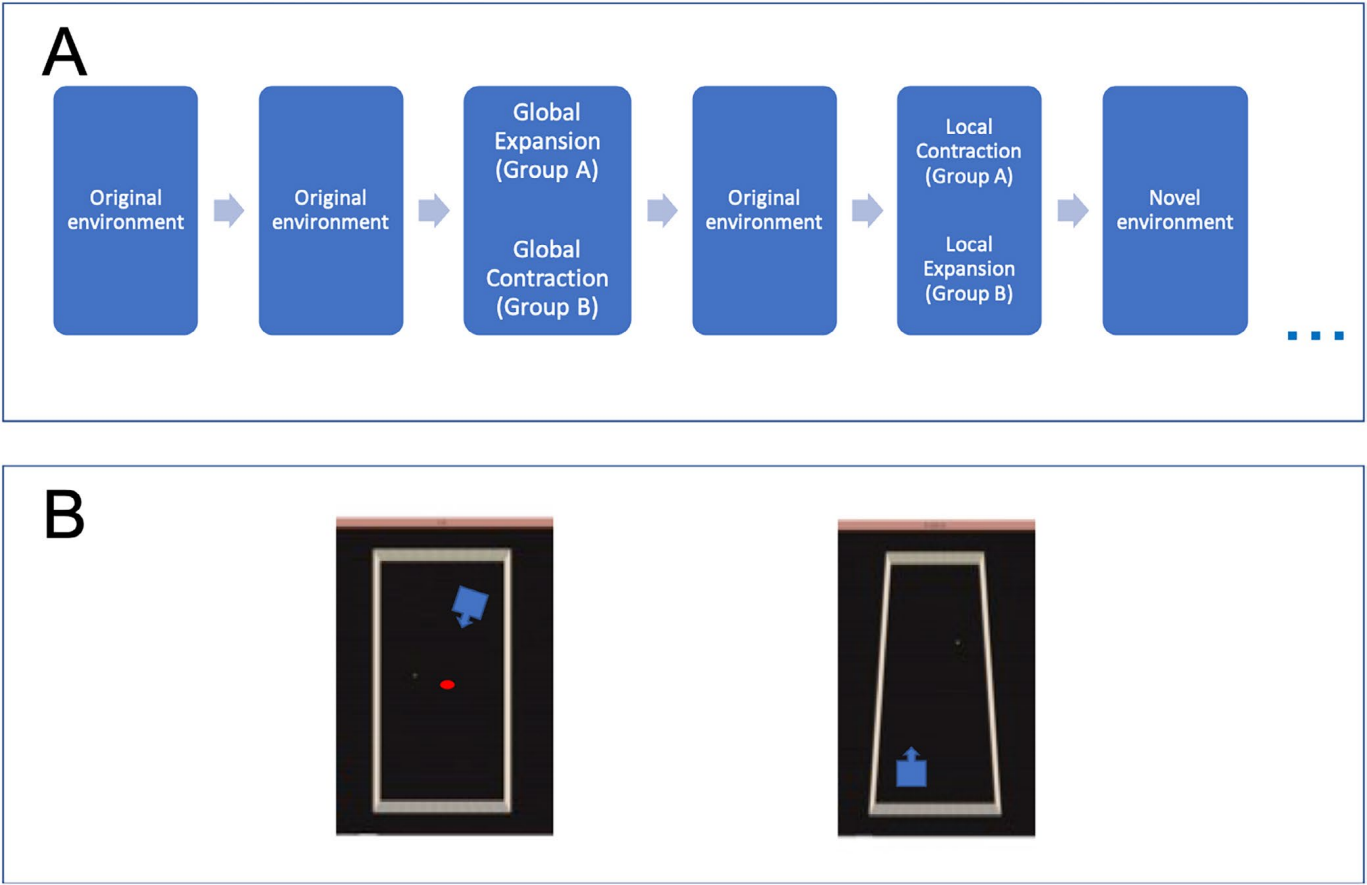
(A) Schematic flow diagram showing the first six trials in the twenty‐two trial experiment. All participants saw the original environment in the first two trials, followed by a global expansion of the original environment for half the participants and a global contraction of the original environment for the other half of the participants. All participants would then complete a trial in the original environment. Half the participants then completed a local contraction trial, whilst the other half completed a local expansion trial. In total, participants completed twenty‐two trials that included, three globally expanded environments (expanded in the vertical axis, the horizontal axis or both axes), three globally contracted environments (contracted in the vertical axis, the horizontal axis or both axes), two locally expanded environments (expanded in the vertical or horizontal axis) and two locally contracted environments (contracted in the vertical or horizontal axis). In addition, participants were occasionally placed in a novel environment (four in total based on Barry et al. (2012)), where the texture and color of the walls, ceiling and floor were changed. (B) Schematic bird's eye view of a local contraction trial. *Left* shows the encoding trial in the original environment, with the blue box indicating the start location and heading direction and the red dot indicates the target object. *Right* shows the retrieval trial in the manipulated environment, with a new start location and heading direction.

Before testing, all participants were exposed to the original environment. This allowed participants to familiarize themselves with the keyboard controls and the general size and appearance of the original environment (texture on the walls and floor). This familiarization phase consisted of an object retrieval task to ensure that participants sampled the whole environment. Participants had 30 s to collect and remember the position of four colored balls (red, blue, green, and yellow) scattered throughout the environment. After the 30 s had elapsed, participants were required to place each ball back in its original location, one after the other. In total, this familiarization phase took approximately 2 min to complete. Once the familiarization phase was complete, testing began. For every test trial, participants completed an encoding phase in the original environment and a retrieval phase in a manipulated environment (Figure 2). In total, participants completed 22 test trials (eight original/no manipulation, three globally expanded, three globally contracted, two locally expanded, and two locally contracted environments, and four novel environments where the floor, ceiling, and wall color and texture were changed).

### 2.5 Encoding Phase

Participants began each encoding phase in the original environment. An arrow suspended above the ground indicated the encoding location that participants were required to walk to. The starting location was randomized across trials. Once the encoding location was reached, participants were pseudorandomly rotated to ensure there was ample distance for the judgment task to occur. Once the rotation was complete, a ball was placed directly in front of participants to indicate the goal location. The ball appeared directly in front of participants to ensure participants were judging distance in one dimension. The distance of the ball was randomized such that the ball could appear anywhere between 5 and 15 m from the location of participants. Once the ball appeared, participants were asked to walk towards it (only the “forward” key was enabled), taking care to remember the distance they traveled.

### 2.6 Retrieval Phase

Participants were transported to one of the manipulated environments in a pseudorandom location and facing direction from a database of 30 locations and directions. These predetermined locations/directions ensured that if the location was less than 15 m from a wall, the heading direction would face away from the wall (allowing space for the participant to recreate the distance). During this transition, a white screen flashed on screen so that participants would not see the loading of the new environment. Participants were asked to recreate the distance they had previously traveled. Again, the controls were limited to just the forward direction. Once they believed they had reached the correct distance, they were required to place the ball down.

The speed of the in‐game character was randomized during the encoding distance and the subsequent distance judgment (selected from a flat distribution, ranging from 0.5 to 2 m/s) to prevent participants from using time alone to estimate distance traveled.

### 2.7 Data Analysis

The error, that is, the accuracy of the participant's distance judgment was calculated by subtracting the encoding distance from the retrieval distance. Error was recorded in two formats: “absolute mean error” and “mean error”. “Absolute mean error” recorded distances irrespective of direction and was used to compare the effect of environmental manipulations (original, novel, global and local) on judgment accuracy. “Mean differences” accounted for direction and were used to investigate whether participants significantly under or overestimated in the expanded and contracted environments.

In total, participants completed eight original, four novel, three globally expanded, three globally contracted, two locally expanded, and two locally contracted trials. Errors from the same condition (with the exception of the novel condition) were averaged.

## 3 Results

### 3.1 Preliminary Analysis

The results of this study came from 51 participants, who were asked to estimate and replicate distances in the following conditions: original environment, novel environment (original environment but with changes to the color and texture of the floor, ceiling and walls), globally expanded environment, globally contracted environment, locally expanded and locally contracted environments (Figures 1 and 2). A preliminary analysis was conducted to investigate whether the order of the trials interacted with the novelty, global alterations or local alterations of an environment on participants' distance judgments. A 4 (manipulation: original, novel, global, and local) × 2 (trial order: expansion first or contraction first) mixed ANOVA revealed no significant effect of trial order *F* (1,49) = 2.76, *p* = 0.103 and no significant interaction between trial order and type of manipulation *F* (3,147) = 0.845, *p* = 0.471. As no significant differences were revealed between participants who saw the expansion first and those who saw the contraction first, data from all participants were combined for all subsequent analyses.

Based on electrophysiological research, it was expected that environmental manipulations (both novelty and rescaling) would influence distance judgments. Compared to the original environment, overestimations in distance judgments were expected in the novelty and the expansion conditions—corresponding to an increase in grid scale. Whereas underestimations in distance judgments were expected in the contraction conditions—corresponding to a reduction in grid scale. Moreover, we predicted that when we compare error in distance judgments across all trials, in the entire environment, error rates in the “global manipulation” condition would be larger compared to error rates in the “local manipulation” condition. This prediction stems from rodent studies showing that, although local manipulations lead to significant grid cell deformations (e.g., reconfigured boundary) (Krupic et al. 2018), these deformations appear to be relatively localized to the immediate proximity of environmental changes. Consequently, we hypothesized that local manipulations would only produce significant errors in trials where the manipulation was in close proximity to the judgment. Trials where judgments were made beyond the altered area would have little impact, resulting in a smaller error rate when averaged across all “local manipulation” trials. Conversely, global manipulations, affecting a larger portion of the environment, were expected to induce more widespread changes in the grid cell firing patterns and thus have a more pronounced effect on distance judgments across the entire space.

**FIGURE 3:**
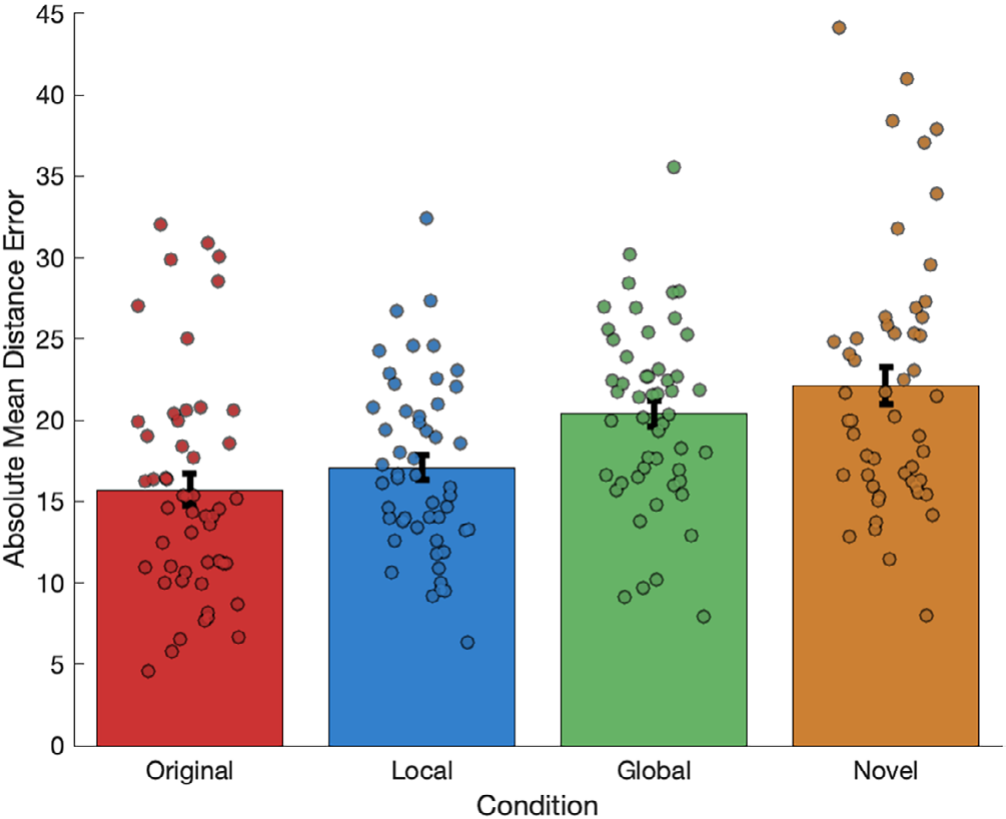
Absolute mean error values (with error bars representing ± SEM) for each environmental manipulation. Manipulations to the context (novelty) and geometry (global and local) of the original environment significantly degraded distance judgments. Changes to the contextual characteristics of the environment degraded distance judgments significantly more than local and global geometric changes. Global geometric changes degraded distance judgments significantly more than local geometric changes.

Environmental manipulations significantly affected participants' distance judgments, using absolute mean error values (repeated measures ANOVA: *F* (3,150) = 20.731, *p* < 0.001, η_p_^2^ = 0.293). Participants exhibited larger absolute distance judgment errors in all manipulated environments compared to the original environment (Figure 3). Post hoc comparisons were conducted using multiple pairwise *t*‐tests, with a Bonferroni correction applied. This analysis revealed that estimates in the original environment were significantly lower than those in novel (*p* < 0.001) and globally altered (*p* < 0.001) environments but not the locally altered environments. Further post hoc comparisons using multiple pairwise *t*‐tests (with Bonferroni correction) revealed that estimates in the locally altered environments were also significantly lower than those in globally altered environments (*p* < 0.01), indicating—as expected—a stronger influence of global geometric changes on participants' judgments compared to local changes. Finally, novel environments elicited a significantly greater effect on distance judgments compared to local (*p* < 0.001) and global (*p* < 0.05) geometric changes, indicating that featural changes elicited stronger effects on distance judgments compared to geometric changes.

**FIGURE 4:**
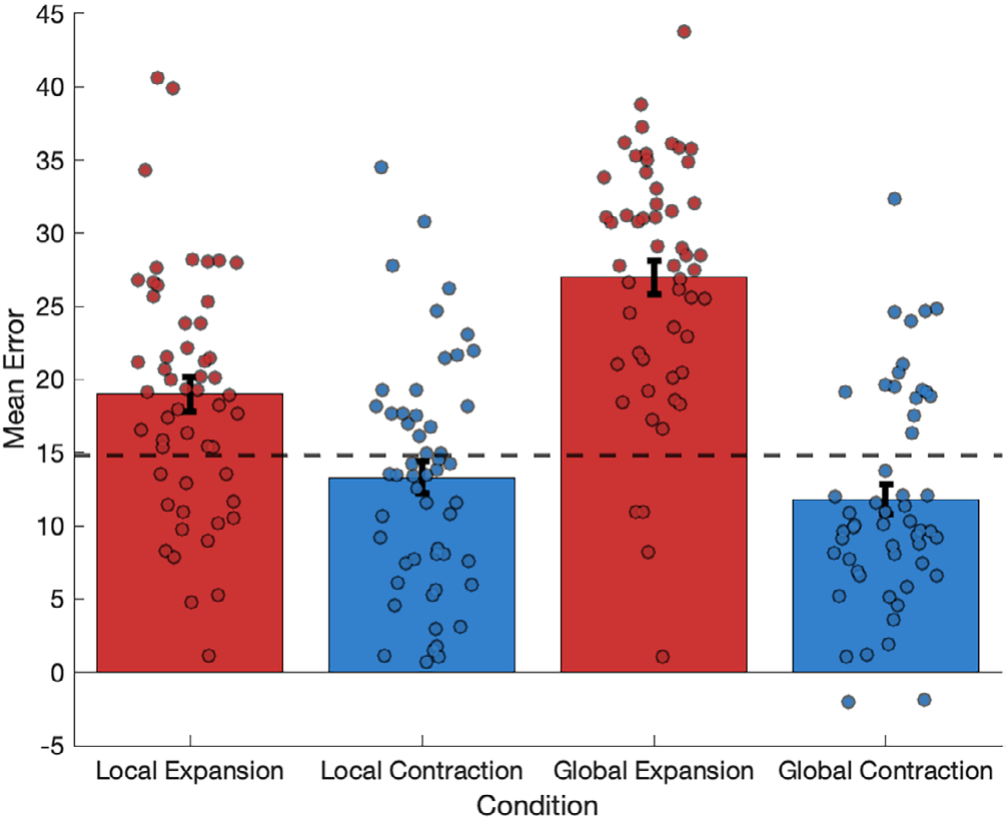
Mean error values (with error bars representing ± SEM) for each geometric manipulation, with a dashed line indicating mean error value of the original condition. The expansion conditions resulted in significant overestimations of distance, with the global expansion producing the greatest overestimation. The contraction conditions also showed overestimations, but these overestimations were smaller than those made in the original condition (dashed line).

A two‐factor repeated measures ANOVA using the mean error scores (rather than absolute mean values to preserve directionality) revealed significant main effects for both “extent” (global/local) (*F* (1,50) = 13.612, *p* < 0.001, η_p_^2^ = 0.214) and “direction” (contraction/expansion) (*F* (1,50) = 97.846, *p* < 0.001, η_p_^2^ = 0.662) of manipulations. Global manipulations induced a significantly greater proportion of overestimations compared to local manipulations (Figure 4). Similarly, Expansion manipulations led to more overestimations than Contraction manipulations. A significant interaction between “extent” and “direction” was also observed (*F* (1,50) = 30.322, *p* < 0.001, η_p_^2^ = 0.378), with the effect of direction being most pronounced during global manipulations. Post hoc comparisons using multiple pairwise *t*‐test (with Bonferroni correction) revealed significant differences between all group comparisons (*p* < 001).

### 3.2 Novelty Across Exposures

A one factor repeated measures ANOVA was conducted on absolute mean error values to determine whether repeated exposure (1st, 2nd, 3rd, or 4th exposure) affected the amount of error in participants' distance judgments within the novel environment. Mauchley's test indicated that the assumption of sphericity is valid *w* (5) = 0.834, *p* = 0.114. The main effect of repeated exposure was not significant *F* (3,150) = 0.893, *p* = 0.446 indicating that, repeated exposure to the novel environment had no effect upon the error in participants' distance judgments, that is, participants did not improve with time.

**FIGURE 5:**
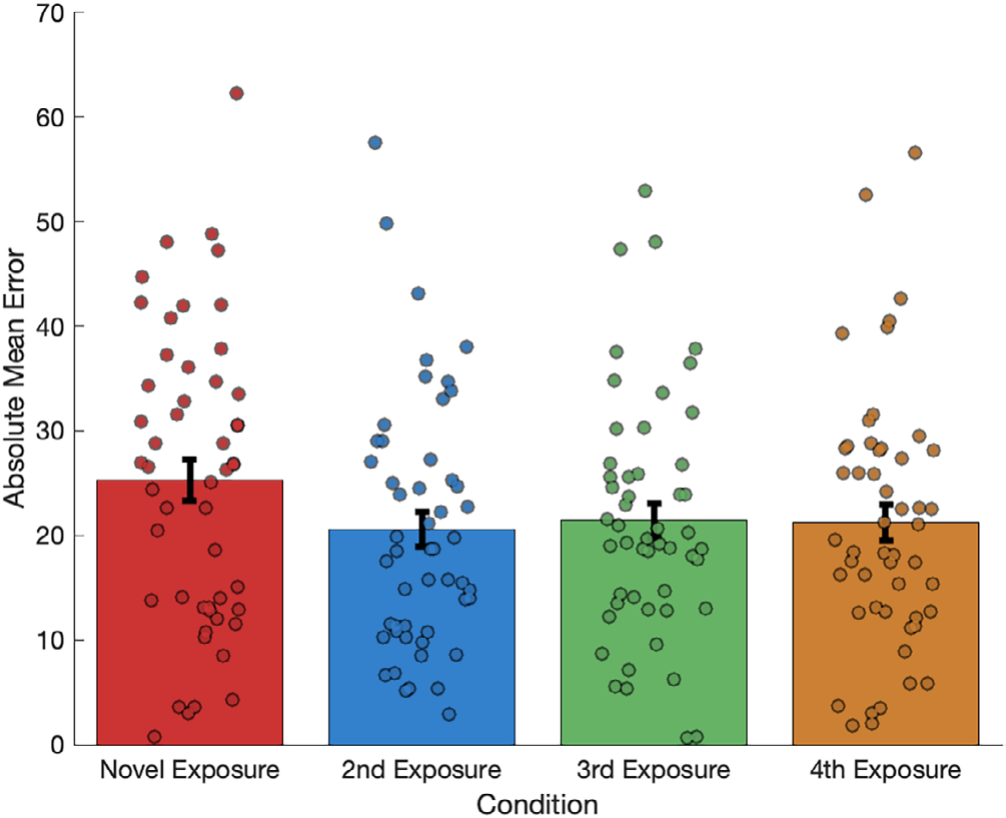
Absolute mean error values (with error bars representing ± SEM) for each presentation of the novel environment. A planned comparison based on previous literature (Barry et al. 2012) showed that the initial novel exposure produced significantly greater distance judgment errors compared to subsequent exposures (mean of the second, third, and fourth exposures) of the contextually different environment.

To further investigate the effect of environmental novelty on distance judgments, we examined how participants' estimations changed between the first and last exposures to the novel environment. Inspired by rodent studies demonstrating rapid grid scale reduction in novel environments (Barry et al. 2012), we hypothesized that novelty's impact on human distance judgments would similarly diminish after initial exposure. To test this, we compared the first novel trial (first exposure) with the fourth novel trial (last exposure). A paired‐samples *t*‐test revealed a significant difference between the first novel trial and the fourth novel trial (*t* (50) = 1.75, *p* = 0.043, η_p_^2^ = 0.245). Participants showed greater error in distance judgments in the initial novel condition compared to the last exposure. This finding suggests that the effect of environmental novelty on distance judgments is most pronounced during the first encounter and diminishes with familiarity (Figure 5).

### 3.3 Global and Local Effects Across Exposures

A two‐way ANOVA on the mean error values was conducted to see if the effect of the global manipulation and the direction of the manipulation (contraction versus expansion) changed over time (three exposures). As before, the main effect of direction was significant, *F* (1,50) = 6.998, *p* < 0.05, η_p_^2^ = 0.123, with expansion manipulations producing a greater error in judgment compared to contractions. There was also a significant effect of time over the three sessions, *F* (2,100) = 36.312, *p* < 0.001, η_p_^2^ = 0.421. A planned comparison found a significant linear effect across time, *F* (1,50) = 69.806, *p* < 0.001, η_p_^2^ = 0.583, where errors were greatest in the earlier manipulations and reduced over time. There was also a significant interaction, *F* (2,100) = 38.284, *p* < 0.001, η_p_^2^ = 0.434.

An additional two‐way ANOVA was conducted to see if the effect of the local manipulation and the direction of the manipulation (contraction versus expansion) attenuated over time (two exposures). As before, the main effect of direction was significant, *F* (1,50) = 14.353, *p* < 0.001, η_p_^2^ = 0.223, with expansion manipulations producing a greater error in judgment. There was no significant effect of time over the two sessions, *F* (1,50) = 1.455, *p* = 0.233. There was no significant interaction, *F* (1,50) = 1.101, *p* = 0.299.

### 3.4 Local Manipulation Effect

We investigated whether local changes to environmental geometry (which would be expected to produce localized effects on grid cells or grid dynamics) differentially impact distance judgments made proximal to the changed region. This analysis helps us understand if the effects of local geometric manipulations on spatial perception are indeed spatially specific, as predicted by grid cell models.

**FIGURE 6:**
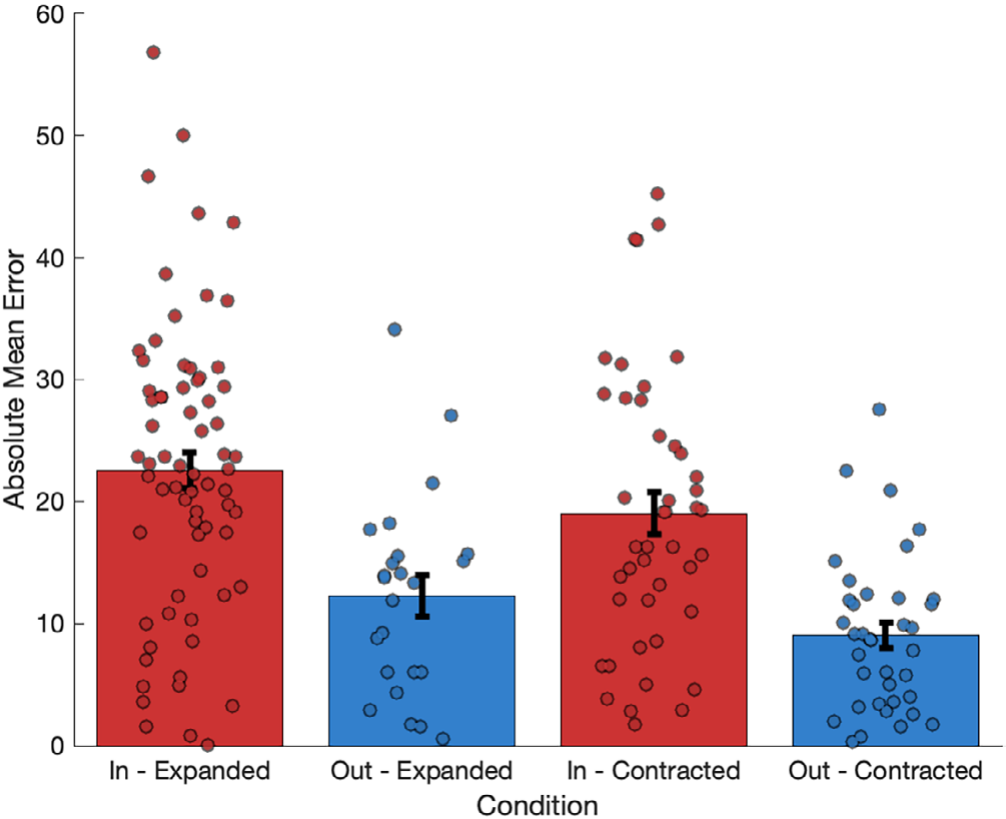
Absolute mean error values (with errors bars representing ±SEM) when the midpoint of the judgment was either inside the manipulated area (red) or outside of the manipulated area (blue) for both local expansion and contraction trials. Judgments made within the manipulated areas were significantly degraded in both expansion and contraction trials compared to judgments made outside of the manipulated areas.

We compared absolute mean error rates for distance judgments made within the area where the local manipulation occurred to those made outside this area (locality). The midpoint of each judgment was used to categorize its location, with midpoints falling in the centre of the environment excluded from the analysis (45 trials). A two‐way independent measures ANOVA found a significant main effect of locality (*F* (1,170) = 35.856, *p* < 0.001, η_p_^2^ = 0.174), where the absolute mean errors of judgments made inside the locally manipulated area were significantly greater than those made outside of the locally manipulated area (Figure 6). As before, locally expanded manipulations produced significantly greater errors than locally contracted manipulations (*F* (1,170) = 3.99, *p* < 0.05, η_p_^2^ = 0.023). There was no significant interaction between the type of manipulation (expansion versus contraction) and the location of the judgment (inside versus outside the manipulated area). We also analyzed absolute mean errors based on whether distance judgments were aligned or misaligned with the direction of expansion/contraction, using a ±45° window to categorize judgment direction. No significant differences were found for any comparison (expansion/contraction in either horizontal/vertical trials).

### 3.5 Variability in Performance

Finally, we investigated the extent of variability in individual performance across conditions, to see how the geometric and contextual effects reported above, influenced performance at an individual level. Pearson's correlations found a significant positive correlation between the original and novel environment (*r* (49) = 0.626, *p* < 0.001); the original and locally manipulated environment (*r* (49) = 0.286, *p* < 0.05) and the original and globally manipulated environment (*r* (49) = 0.549, *p* < 0.001). These findings suggest that participants who were performing well in the original condition (i.e., “good performers”), maintained this relative standing in the other conditions. Moreover, this relative performance was also consistent across manipulation “direction”, where Pearson's correlations found a significant positive correlation between the global contraction and global expansion environment (*r* (49) = 0.628, *p* < 0.001) and the local contraction and local expansion environment (*r* (49) = 0.724, *p* < 0.001). These findings further indicate that a participant's relative performance was consistent across the experimental manipulations.

## 4 Discussion

Our study demonstrates that manipulations of environmental context and geometry significantly influence human distance judgments, with effects varying based on the nature and extent of the manipulation. Contextually manipulated environments elicited the highest degree of error in participants' distance estimates, followed by manipulations to the global geometry of the environment. We observed that global manipulations produced larger changes in distance judgments than local changes, aligning with known grid cell responses: localized environmental alterations typically affect grid cells only in the vicinity of the change, while global manipulations have a more widespread impact on the grid cell network (Krupic et al. 2015; Stachenfeld et al. 2017; Stensola et al. 2015).

Specifically, our analysis revealed significant main effects for both the extent (local vs. global) and direction (expansion vs. contraction) of manipulations, as well as an interaction between these factors. Expanded environments induced greater overestimations compared to contracted environments, supporting the view that changes in grid cell firing patterns correspond to changes in distance judgments. Surprisingly, the contracted environments produced overestimations as well as underestimations in error distance judgments. This tendency for participants to overestimate distance was also seen in the original environment, where no manipulations took place. Although the global contraction and local contraction conditions did not differ compared to the original condition, the presence of overestimations in the contracted environments was contrary to predictions. This bias towards overestimations of distance in desktop virtual reality environments has been found previously (Guzsvinecz et al. 2023), with subjects over‐walking distances to goals (Kang et al. 2023) and may be linked to variables such as available field of view (Kline and Witmer 1996). Our subjects consistently overestimated distances in the novel environment, an effect that largely attenuated after a single exposure—a pattern that directly parallels the transient grid scale expansion observed in rodents encountering novel environments (Barry et al. 2012).

Taken together, these findings suggest a hierarchical impact of environmental manipulations on spatial cognition, with contextual and global geometric changes exerting the strongest influence, followed by local geometric alterations. Notably, this pattern aligns with predictions derived from grid cell electrophysiological studies in rodents, which have reported differential effects of Novelty, global, and local environmental changes on grid cell firing (Barry et al. 2007, 2012; Stensola et al. 2015). Our results also reflect predictions from computational models describing how grid cell firing is anchored to and affected by local cues, and how changes to grid cell firing can generate errors in navigational vectors calculated from deformed grids (Banino et al. 2018; Burgess et al. 2007; Bush et al. 2015; Carpenter and Barry 2016; McNaughton et al. 2006). Furthermore, in the local manipulation conditions, changes to the distance judgments appear to be localized, where judgments were significantly greater in the manipulated area compared to those judgments made outside of the manipulated area. These findings suggest that environmental manipulations produce localized distortions in human spatial representations, with effects concentrated near the altered regions rather than distributed uniformly across space.

The consistency between our behavioral findings and these neurophysiological and computational predictions suggests possible parallels in spatial processing across species. However, it is important to note that behavioral data alone cannot directly demonstrate grid cell involvement, and multiple neural mechanisms likely contribute to human distance estimation, including contributions from hippocampal place cells, retrosplenial cortex, parietal cortex, and visual areas. While our results are consistent with predictions derived from grid cell research, they may equally reflect the operation of other spatial processing systems or a combination of multiple mechanisms.

Regardless of the specific neural mechanisms involved, our findings shed light on the influence of geometric and contextual (or featural) cues during navigation. During the process of disorientation, animals and humans tend to rely on geometric cues over featural cues to reorient (Batty et al. 2009; Lee and Spelke 2010), supporting the idea of a geometric module (Cheng 1986). Conversely, when humans and animals do not undergo disorientation, featural cues may instead have greater influence over navigational behaviors (Knight et al. 2011; Lourenco and Huttenlocher 2006). The present findings go some way to supporting these findings, as changes to contextual cues had a greater impact on distance judgments compared to geometric changes in non‐disoriented participants.

Broadly these results can be interpreted through a simple “model” of grid cell‐based distance estimation. In this model, distances experienced during the encoding phase are represented and stored as vectors, or the magnitude of those vectors, in grid space. During the response phase, the distance traveled is directly compared to this stored representation. Consequently, any change in grid cell spacing will result in a proportional error in distance estimation. For instance, consider a subject traversing 3 m during encoding, which corresponds to crossing three fields of a 1 m‐scale grid cell network. If environmental novelty causes the grid scale to expand to 1.5 m, the subject would need to travel 4.5 m during retrieval to cover the same distance in grid space. Such a framework provides one possible mechanistic explanation for how changes in environmental geometry or novelty can systematically bias distance judgments. While this grid cell‐based model aligns with our findings of overestimation in expanded and novel environments, similar predictions could arise from other spatial coding schemes or from distributed changes across multiple spatial processing systems.

Changing the context of the environment (adding novel wall, ceiling and floor colors and textures) led to a greater overestimation of distance judgments, which supports previous findings (Barry et al. 2012). Furthermore, the overestimations seen in the first novel trial were significantly greater than those in subsequent trials, which again supports Barry et al. (2012). However, unlike this previous study, no significant differences were found between the subsequent novel trials (2nd, 3rd, and 4th novel trials). This may have resulted from the different methodologies employed. Barry et al. (2012) subjected rats to the novel environment in three consecutive rather than alternating instances. Participants in the current study may not have had enough continuous exposure to become habituated to the novel environment. This would explain the general trend towards being more accurate, but not a significant effect of repeated exposure. Secondly, it may be possible that participants needed more exposure time in each novel exposure. Barry et al. (2012) exposed rats to each environment for 20 min compared to 30 s for participants in the current study.

A primary limitation of this study is the indirect nature of our inferences about neural mechanisms. While our behavioral results align with predictions based on rodent grid cell studies, we did not have direct access to participants' neural activity during the task. Consequently, we cannot definitively attribute the observed effects to grid cell activity specifically. As noted above, human distance estimation likely involves multiple brain regions and cell types beyond grid cells, including hippocampal place cells, boundary cells, head direction cells, and cortical areas involved in visual processing and spatial memory. Our conclusions about the relationship between environmental manipulations and neural function are necessarily speculative and based on extrapolations from rodent electrophysiology and computational models. The behavioral patterns we observe could arise from any number of spatial processing mechanisms or their interactions, not exclusively from grid cell dynamics. This underscores the need for future research that combines human behavioral tasks with neuroimaging techniques. Functional MRI studies have already provided evidence for grid‐like representations in the human entorhinal cortex (Doeller et al. 2010; Jacobs et al. 2013) and have shown that these representations can be modulated by environmental boundaries. While less common, magnetoencephalography (MEG) has also been used to investigate grid‐like neural codes in humans (Staudigl et al. 2018). These non‐invasive techniques could be adapted to our paradigm to more directly link behavioral changes in distance estimation to alterations in grid‐like neural patterns.

Moreover, it is worth noting that direct recordings of human grid cells are possible in patients with intracranial electrodes implanted for clinical purposes (Jacobs et al. 2013). While such opportunities are rare, replicating our study or a similar paradigm with participants who have indwelling electrodes could provide the most direct evidence of how environmental manipulations affect human grid cell activity and corresponding distance judgments. Despite this limitation, our study's strength lies in its systematic manipulation of environmental context and geometry, closely mirroring paradigms used in rodent grid cell research. This approach allows for a more direct comparison between human behavior and rodent neurophysiology, providing a valuable bridge between these two domains of spatial cognition research.

This study advances our understanding of human spatial cognition, demonstrating behaviorally that environmental manipulations affect distance judgments in ways consistent with grid cell function. Our findings bridge rodent and human spatial navigation research while offering insights into how the brain computes and represents spatial information more broadly. Future investigations could extend this paradigm to clinical populations with spatial memory deficits, potentially yielding valuable insights into various neurological conditions. The principles unveiled here might also inform the development of more intuitive navigational aids or virtual reality environments. Continued probing of the links between neural representations and spatial behavior will move us closer to a comprehensive model of spatial cognition that spans from cellular mechanisms to real‐world navigation.

## Funding

The authors have nothing to report.

## Data Availability

The data that support the findings of this study are available from the corresponding author upon reasonable request.
